# Effect of Resistant Starch Sources on the Physical Properties of Dough and on the Eating Quality and Glycemic Index of Salted Noodles

**DOI:** 10.3390/foods11060814

**Published:** 2022-03-12

**Authors:** Po-Hsien Li, Chien-Wen Wang, Wen-Chien Lu, Yung-Jia Chan, Chiun-Chuan Roger Wang

**Affiliations:** 1 Department of Food and Nutrition, Providence University, 200, Section 7, Taiwan Boulevard, Shalu District, Taichung City 43301, Taiwan; pohsien0105@pu.edu.tw (P.-H.L.); or ustar12@gmail.com (C.-W.W.); 2 U-Start Co., 109, Shenan St., Shengang Dist., Taichung City 42944, Taiwan; 3 Department of Food and Beverage Management, Chung-Jen Junior College of Nursing, Health Sciences and Management, 217, Hung-Mao-Pi, Chia-Yi City 60077, Taiwan; m104046@cjc.edu.tw; 4 College of Biotechnology and Bioresources, Da-Yeh University, 168, University Rd, Dacun, Changhua 51591, Taiwan; chanyungjia@gmail.com

**Keywords:** composite flours, resistant starch, salted noodles, cooking time, textural properties, estimated glycemic index, sensory attributes

## Abstract

The aim of this study was to evaluate the characteristics and eating quality of salted noodles that are incorporated with different formulations of flour. Up to 20% of wheat flour was substituted by composite flours of highly resistant starches, including heat moisture treatment corn starch (HMT-CS), high-amylose corn starch (Hylon VII), and green banana flour (GBF). The physical properties of dough, in conjunction with the eating quality and estimated glycemic index (EGI) of cooked salted noodles, were investigated in this study. The results concluded that the incorporation of GBF, HMT, and Hylon VII not only affected the water absorption and mixing tolerance of the dough, but also the maximum resistance to extension and extensibility in terms of the extensographic properties. Meanwhile, GBF, HMT, and Hylon VII incorporation significantly increased the resistant starch content and decreased the fat content of the noodle samples. The textural profile analyses of cooked salted noodles indicated that hardness, gumminess, chewiness, and shearing force increased; nevertheless, springiness declined with the increase in the proportion of flours from 10 to 20%. The sensory evaluation detected that wheat flour composited with 10% GBF and HMT flours could produce acceptable quality noodles as compared with normal typical control noodles. In the meantime, salted noodles incorporated with GBF, HMT-CS, and Hylon VII flour decreased the estimated glycemic index (EGI) dramatically. The result of this study concluded that incorporation of various sources of resistant starch flour could develop a low-GI noodle with good acceptability that may contribute to gastrointestinal health.

## 1. Introduction

The evolution of valued products has recently been a deliberate focus of the food industry. Consumers are challenging foods in terms of their traditional nutritional requirements, whereas additional health benefits, such as in nutraceutical foods, are anticipated from regular intake. Rich sources of resistant starch (RS) have commonly been applied in commercial food manufacturing in the last decades. RS is known as an important contributor to the maintenance of gastrointestinal health. It is recognized that incorporation of resistant starch in staple foods could be considered in order to increase the daily dietary fiber intake [1]. Various studies have demonstrated that RS is a part of dietary fiber, which is explicated as the sum of starch from the fraction of starch degradation that outflows from digestion in the human small bowel (the duodenum, jejunum, and ileum) and reaches the large intestine where resident microflora carry out the process of fermentation. The resultant short chain fatty acids (SCFA), as well as acetate, propionate, butyrate, isobutyrate, valerate, and isovalerate, are produced by the fermentation of microflora, playing an important role in metabolization and lowering the risk of pathogen overgrowth in the large intestine [2]. RS plays a potential role in the avoidance of coronary heart disease, diabetes, and obesity [3]. A high-RS diet also amplifies apoptosis in the intestine, consecutive to externally causing gene damage, which proposes the capability of RS to regulate the emergence of colonic cancer [4].

Asian noodles are a commonly eaten food, including udon and ramen noodles (Japanese white salted noodles and Korean white salted noodles, respectively), as well as Chinese yellow alkaline noodles (Hokkien noodles). The rheological properties of salted noodles are closely correlated with the compositions of wheat flour used, a factor that has been broadly examined [5,6]. Due to the consumer’s demand for food with additional health benefits, use of an RS-rich composited flour could be a vital constituent of a healthy diet in the preparation of noodles. There is a novel propensity to augment dietary fiber, such as RS, to the elementary ingredients, namely, wheat flour for noodle preparation, in order to provide an additional health benefit during its regular digestion [7].

Banana is one of the most imperative climacteric fruits in Taiwan, being a potential source of starch, and has value in terms of investigation due to its abundance, predominantly in tropical countries. Unripe or green banana starch is extremely impervious to digestion in rats and humans [8]. Previous studies also demonstrated the structural features of RS from green banana, exhibiting that the banana starch effectively reduced the in vivo digestibility [8]. Moreover, the green banana flour that is produced from industrial production also has gained interest in view of its nutritional value, specifically its high amount of resistant starch content (approximately 40.9–58.5%) [9] and dietary fiber (6.0–15.5%) [10], yet its bioactive compounds, for example, phenolic acids, show high antioxidant capacity [11].

A diet with high-amylose corn starch (Hylon VII, with 70% amylose content) and heat moisture treatment corn starch (HMT-CS) also contains a significant amount of RS, up to 46–50% of which can be offered as a nutraceutical food to satisfy consumer demand [3,12]. Heat-moisture treatment (HMT) is a hydrothermal method wherein the starch granules of low moisture content (18–30%) compounds are heated up for a few hours at a comparatively high temperature, between 90 and 120 °C [13]. Moreover, previous studies exhibited that heat-moisture treatment (HMT) upsurged the resistant starch from 7.02% to 18.04% [14]. Furthermore, HMT was dramatically lower down the peak viscosity and setback viscosity of the starch, and remarkably postponed the beginning of gelatinization, peak, and conclusion temperatures [14].

Nevertheless, to the best of our knowledge, no such material has been found to date regarding incorporation of HMT corn starch, Hylon VII corn starch, and green banana flour (GBF) to salted noodles. Hence, the goal of this current study was to apply several sources of RS-rich flours, such as HMT corn starch, Hylon VII corn starch, and green banana flour (GBF), to formulate a composited flour for dough and salted noodle preparation. The physicochemical properties, sensory quality, and glycemic index of the salted noodles were also evaluated in this study.

## 2. Materials and Methods

### 2.1. Flour Formulation

The flour composition in this current study was formulated with different proportions of RS source flours, including the HMT, Hylon VII, and GBT (0, 10, 20%), together with the wheat flour (Chi-Fa Enterprise Co., Taichung, Taiwan). All chemicals used were American Chemical Society (ACS)-certified chemical grade.

#### 2.1.1. Green Banana Flour (GBF)

Green banana (Musa padisiaca) was locally grown at a local farm at Nantou, Taiwan. Green banana was treated by the procedures of washing, peeling, dicing, and being air-dried by a hot air oven. The dried green banana was intermixed by a blender, generating a flour with consistent size by passing through a 100-mesh, which is 150 µm of sieve. The green banana flour (GBF) was put into a polyethylene zipper bag and stored in a freezer until further use.

#### 2.1.2. Heat Moisture Treatment Corn Starch (HMT-CS)

The moisture content of corn starch was guided to around 30% before further treatment. The samples were heated in a hot air oven at a temperature of 100 °C for a continuous 16 h. After the flour cooled down, the starch was air-dried to assure consistent moisture content (10%) [15,16].

#### 2.1.3. High-Amylose Corn Starch (Hylon VII)

High-amylose corn starch, namely, Hylon VII, was supplied by the National Starch and Chemical Company (Bridgewater, NJ, USA). The sample was stored in an air-tight container before further use.

### 2.2. Proximate Analyses

The proximate analyses for moisture, crude protein (method 46–12; N × 5.37 for wheat flour), and ash content (methods 08–01 and 08–17) of the wheat flour and salted noodles were accomplished in accordance with the method of the AACC (2000). Crude lipid (Method 30–25) was analyzed by using the Soxhlet method, while the crude fiber (method 32–20) was measured gravimetrically after chemical digestion and solubilization. The contents of moisture, crude protein, and ash of the wheat flour were 13.04, 10.98, and 0.39%, respectively.

### 2.3. Preparation of Salted Noodles

We added 2% of salt into the formulated flours. Next, mixing was carried out at low speeds with water for 5 min in a mixer, and it was continued by rolling into a dough sheet with around 3 mm of thickness. Folding and sheeting were consequently carried out. The dough sheet was put aside to rest for around 1 h, and it was passed through the sheeting rolls 3 times, gradually decreasing the gaps of the roll from 2.60 to 2.30 to 2.00 mm. The noodle strand was cut uniformly from the noodle sheet, and the organized noodles were stored in an air-tight container [17].

### 2.4. Dough Characteristics

A Farinograph (E-380, Brabender OHG, Germany), by referring to the standard method of AACC 54-21 (2000), was applied to investigate the rheological properties of the dough sample. Different formulations of HMT, Hylon VII, and GBT, at 0, 10, and 20%, respectively, together with wheat flour, were prepared before the examination. During the examination, the water absorption, arrival time, departure time, dough stability, peak time, and mixing tolerance index were investigated. Meanwhile, the flexible properties of the dough with different amounts of HMT, Hylon VII, and GBT were also measured by using an Extensograph (Brabender, Duisburg, Germany), which was in reference to the standard method of AACC 54-10 (2000). After Farinograph measurements, the dough was cut into two parts and passed through the balling, followed by the mold unit of the Extensograph. The dough was rested for 45 min in the fermentation cabinet and was followed by stretching. The resistance to extension (BU) and extensibility (cm) were the parameters included in the test.

### 2.5. Salted Noodles Evaluation

#### 2.5.1. Physical Properties

A total of 100 mL of boiling water was prepared, and 10 g of salted noodles was cooked in for 5 min and drained. The cooked noodles were dried in a hot-air oven at a temperature of 115 °C, and the leftover solids were measured to calculate the cooking loss, which was stated as a percentage of the initial dry matter.

Cooking weight gain (%) = (final weight − remaining solid weight)/initial weight of noodles

Cooking weight loss (%) = 100% − (remaining solid weight/initial weight of noodles)

#### 2.5.2. Texture Profile Analysis

The texture profiles of the cooked salted noodles were evaluated by a texture analyzer, with the TA-XT2 model, equipped with Stable Micro Systems. First, the noodle was cooked in 500 mL of boiling distilled water for 5 min, and then cold water was used to rinse the noodles for 1 min. Next, the cooked noodle sample was held by tension grips about 3 mm apart in the TA-XT2i and was extended at 0.8 mm/s until failure or breakdown. The maximum slope and the decrease in force on breaking was verified from the force (g)–displacement (mm) curve. The determination of tensile strength of the cooked noodle sample was carried out with modification [18].

Five 6 cm long strands were arranged perpendicularly to the pasta blade probe on a flat aluminum platform base in order to determine the cutting force. The probe moved down through the noodle sample at a speed of 1 mm/s until it touched the platform base. The test was performed twice at different locations to acquire an average result.

For the TPA study, six replications of the noodle sample were examined within 3 min after cooking and cooling. The noodles were positioned on a flat aluminum platform base, and a 3 mm diameter steel cylinder probe crushed the noodle sample to 70% of its original height at a speed of 2 mm/s. The characteristic of the noodle samples in terms of hardness, cohesiveness, springiness, gumminess, and chewiness were recorded from the force-time curves.

#### 2.5.3. Sensory Evaluation

Twelve panelist members made up of graduate students of the Food and Nutrition Department at Providence University were selected to be involved in the sensory evaluation of noodles with different formulations of HMT, Hylon VII, and GBT. Rating test training was carried out on the panelists to assure the panelists evaluated samples properly. A multi-sample comparison test was applied to assess the color, stickiness, cohesiveness, firmness, and overall acceptability of the noodle samples. The multi-sample comparison test of cooked noodles was assessed on a 9-point scale with 1 representing “extremely low” and 9 representing “extremely high”. The color of cooked noodles was evaluated in terms of whiteness on a 9-point scale, with 1 representing “extremely light” and 9 representing “extremely dark”. The overall acceptability test of cooked noodles was estimated on a 9-point scale, with 1 as the “lowest” and 9 as the “highest”. A hedonic scale was applied to evaluate the overall acceptability. The noodle samples were cooked in boiling distilled water for 5 min and cooled in drinking-grade tap water for 1 min before serving. Samples were served individually in a randomized order on plastic trays. Panelists were requested to rinse their mouths with deionized drinking water in the middle of testing each sample.

### 2.6. Total Starch and Resistant Starch

Ground samples (100 mg) were placed in 2 mL of 2 M KOH and were continuously stirred in an ice/water bath for 20 min. To the solubilized starches, we added 8 mL of 1.2 M sodium acetate buffer (pH 3.8) and hydrolyzed them using 0.1 mL of α-amylase and 0.1 mL of amyloglucosidase (Megazyme International, Ireland). The mixture was mixed up thoroughly and incubated at 50 °C in water baths for 30 min. After centrifugation, the glucose content in the supernatant was measured by using a glucose oxidase-peroxidase (GOPOD) kit. Absorption was measured at a wavelength of 510 nm, and glucose concentration was converted into starch content by using a factor of 0.9. The TS content was converted from the content of glucose [19].

A Megazyme Resistant Starch Assay Kit (AOAC Method. 2002.02) was used to measure the resistant starch (RS) content. Ground samples (100 mg) were hydrolyzed by 40 mg of α-amylase solution at 37 °C for 16 h. The hydrolysates were centrifuged, and the supernatants were removed. The residues were analyzed for starch as described above using amyloglucosidase from Aspergillus niger (Megazyme, Wicklow, Ireland).

### 2.7. In Vitro Starch Digestibility and Estimated Glycemic Index

In vitro starch digestibility in salted noodles was evaluated by using the method of Reshmi et al. (2020) with modifications [19]. A total of 100 mg of salted noodles was homogenized with 10 mL HCl-KCl buffer (pH 1.5). Next, 0.2 mL solution containing 1 mg of pepsin (from porcine gastric mucosa, Merck, Kenilworth, NJ, USA) in 10 mL of HCl-KCl buffer was added to the sample, followed by incubation at 40 °C in a water bath for 1 h. The volume was diluted to 25 mL with tri-maleate buffer (pH of 6.9). A total of 5 mL of Tri-maleate buffer that contained 2.6 IU of α-amylase (A-3176, Sigma, St. Louis, MO, USA) was added to each sample. Samples were incubated at 37 °C in a water bath for 180 min. A 1 mL aliquot sample was taken out from each tube every 30 min. The aliquots of α-amylase were inactivated immediately in 100 °C boiling water for 5 min and then refrigerated. Then, 3 mL of 0.4 M sodium acetate buffer (pH 4.75) was added to each aliquot, and 60 µL of amyloglucosidase (Megazyme International, Wicklow, Ireland) was used to hydrolyze the digested starch into glucose after incubation at 60 °C in a water bath for 45 min. Glucose concentration was determined by using a glucose oxidase-peroxidase (GOPOD) kit. The glucose was converted into starch by multiplying it by a factor of 0.9. The rate of starch digestion was reported as a percentage of the total starch hydrolyzed at different times, namely, 30, 60, 90, 120, 150, and 180 min.

The first order equation had the form C = C ∞ (1 − e^−kt^), where C corresponds to the percentage of starch hydrolyzed at time t, C∞ represents the percentage of starch hydrolyzed after 180 min, k represents the kinetic constant, and t represents the time (min). The parameters C∞ and k were assessed for each type of starch and each formulation on the basis of the data obtained from the in vitro hydrolysis procedure. The areas under hydrolysis curves (AUC, 0–180 min) were calculated. The percentage of total glucose release from each sample was calculated as the hydrolysis index (HI) to that of glucose released from white bread. The estimated glycemic index was calculated according to the equation of EGI = 39.71 + 0.549HI.

### 2.8. Statistical Analysis

Each analysis and data value is reported as mean value and standard deviation. All data were examined by using the ANOVA test. The F-value was significant when *p* < 0.05 in the ANOVA analysis. Meanwhile, the treatment means were calculated and compared by the Duncan’s new multiple range test.

## 3. Results and Discussion

### 3.1. Farinograph Characteristics

The farinographs for the composited flour blends are indicated in Table 1. Addition of the resistant starch sources improved the differences in the dough’s mixing behavior, as analyzed by the farinograph. Determination of the farinograph characteristics of dough is crucial in measuring and recording the mechanical strength of the dough during processing, allowing for the achievement of optimal flour mixtures in order to gain good-quality final products [20]. The results show that the supplementation of resistant starch sources mostly affected the water absorption, particularly in flour with higher resistant starch content. Hence, the results of this study showed that addition of resistant starch mainly increased the water absorption properties [21]. The highest absorption was found with the addition of 20% Hylon VII, followed by HMT starch and GBF. This is possibly caused by the excessive number of hydroxyl groups present in the structure of resistant starch, which allow for more water connections through hydrogen bonding [22]. The indicators of dough strength, for example, the dough development time (DDT), breakdown time, stability, and peak time values, with higher values signify stronger dough. The resistant sources did not affect the DDT of dough but decreased the breakdown time and stability with the increase in the proportion of resistant sources in the blended flour. Previous studies specified that a higher proportion of wheat bran in formulated flour echoes a lower gluten content, which means higher DDT, extending the time to hit the maximum consistency of dough, and therefore enhancing the dough stability [23]. Thus, DDT has been robustly linked with the flour’s water absorption percentage [24]. Rising in resistant starch source proportions from 0 to 20%, particularly in Hylon VII corn starch, caused the breakdown time of dough to be reduced. The dough of wheat flour demonstrated the highest dough stability. When the proportion of resistant starch source flour in the composite flour was increased, it caused the stability of dough to decline. Furthermore, the results clearly established that strong gluten flour contributed the highest stability value in the wheat flour group, but the stability was affected by the composited flours because of lower gluten ratios of dough. A previous study involving blended wheat flour and soy flour also detected the same trend for dough stability [25]. Moreover, for the mixing tolerance index (MTI), the results showed a uniformity difference of dough between the height at the peak and the height after 5 min. A huge effect was detected in the MTI result, while upsurge in the proportions of the resistant sources flour involved, particularly in Hylon VII corn starch (38.0 B.U. for 10% Hylon VII, and 63.5 B.U. for 20% Hylon VII), an increase in the MTI of the dough (25.0 B.U. for 10% GBF, and 45.5 B.U. for 20% GBF; 32.0 B.U. for 10% HMT, and 56.5 B.U. for 20% HMT). These phenomena were due to the circumstance of there being less gluten present in the formulated flour, causing a lower interaction between starch and gluten, therefore resulting in higher MTI.

### 3.2. Extensograph Measurements

The effects of various additions of resistant starch source flours towards the extensographic properties of composite flour at different resting times are exhibited in Table 2. The maximum resistance to extension (B.U.) increased between the resting time of 45 and 90 min, with the highest upsurge between 45 and 90 min. The maximum resistance to extension raised up for all samples, including the control sample (58 B.U. to 743 B.U.), as the inclusion of resistant starch source flour increased from 0 to 20%. This result could have been due to the connection between polysaccharide and protein in the flour [26,27]. Nevertheless, addition of GBF, HMT, and Hylon VII corn flour increased in the proportions, also causing the extensibility of dough to decrease. The replacement of wheat flour by resistant starch source flour resulted in the reduction of gluten formation and the weakening of the binding structure of the dough. Furthermore, the current result of this study also corresponded with previous studies, who exhibited that increases in chickpea or cereal bran proportions could lower the extensographic extensibility values of the dough [27,28].

### 3.3. Proximate Compositions, Total Starch Content, and Resistant Starch Content of Cooked Salted Noodles

Table 3 demonstrates the proximate compositions, total starch content, and resistant starch content of cooked salted noodles containing different proportions of resistant starch source flours. Additional resistant starch source flour appreciably affected the resistant starch content in this study. Resistant starch measurements of noodles without RS source flour added was 3.25%, and 14.12%, 10.92%, and 7.27% for noodles made with 20% Hylon, 20% GBF, and 20% HMT-CS, respectively. Consequently, RS in corn flour by HMT increased the RS content of salted noodles upon incorporation, which complied with the findings of the previous study [29]. Added green banana flour (GBF) was also led to significant amounts of crude fiber, namely, 2.15% and 2.31% for the samples 10% GBF and 20% GBF, respectively. Nevertheless, crude fats and crude proteins were substantially lower in all the noodle samples. Lower crude fat and crude protein contents in salted noodle samples might have been due to dilution contributed to by the high-water holding capacity of resistant source flour used [30].

### 3.4. Texture Profile Analyses

The texture profiles of noodle samples that were prepared from different proportions of resistant starch source flours are presented in Table 4. The incorporation of GBF increased the hardness (627 to 683 g × force), cohesiveness (0.56 to 0.58), gumminess (351.1 to 396.1 g × force), chewiness (326.5 to 352.6), and shearing force (201.6 to 239.7 g × force); however, it lowered the springiness from 0.93 to 0.89 with the increases in the GBF proportions from 0 to 20%. A similar trend was observed for noodles with HMT incorporation, except the cohesiveness decreased from 0.62 to 0.56 for noodle samples with 10 and 20% HMT, respectively. The current result agrees with the findings of Li et al. (2012), who described that the force required to compress was raised up in directional proportion with the yam flour in the dough [17].

### 3.5. Cooking Quality of Salted Noodles

Table 5 shows the cooking weight gain and cooking weight loss of noodles containing different proportions of resistant starch source flours. Incorporation of resistant starch flour at 10 and 20% decreased the cooking weight gain for all noodle samples; on the other hand, it increased the cooking weight loss. There was no significant difference between the cooking weight gain for HMT samples and Hylon VII samples, nor in the cooking weight loss for all samples tested, except the control sample. The difference in the gluten fraction was primarily associated with the cooking quality of the sample. The addition of resistant starch flour causes the dilution of the gluten fraction, hence decreasing water retention in the noodle samples [31].

### 3.6. Sensory Evaluation

**Figure 1:**
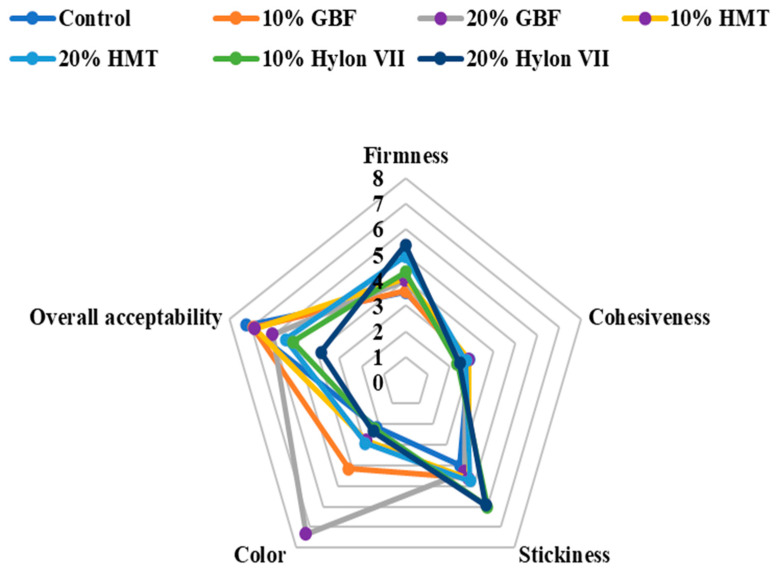
Sensory profiles of cooked salted noodles containing different proportions of resistant starch source flour.

Sensory evaluation or testing was carried out to evaluate the perspective of the consumers, being the most dependable test to develop a product that meets consumers’ needs. The sensory evaluation of noodles with the addition of resistant starch flour is presented in Figure 1. In terms of the color of the cooked salted noodles, the sample that was prepared from 10 to 20% GBF appeared to have a significantly higher brown color in comparison with the other noodles due to the enzymatic browning reaction of the green banana during flour preparation. Nonetheless, the 20% GBF noodles sample was at the top in terms of the score of the color attributes. There was no significant difference in cohesiveness among the control, green banana flour, and HMT flour samples. The salted noodles containing 10% green banana flour and 10% HMT flour received the same overall acceptability scores as the controls. This result showed that by using the green banana flour and HMT corn flour as a proportional replacement for wheat flour, up to 10% could gain comparable quality and a nice appearance relative to normal typical salted noodles.

### 3.7. In Vitro Starch Digestibility and Estimated Glycemic Index

**Figure 2:**
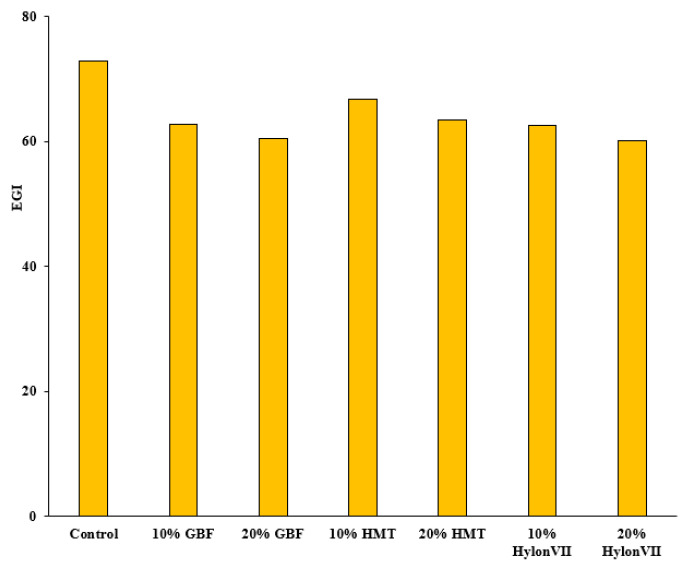
Effect of different proportions of resistant starch sources on the estimated glycemic index (EGI) of salted noodles.

The hydrolysis index of salted noodles containing resistant starch source flour was decreased with the increases of the proportion of resistant starch source flours. The lowest hydrolysis index was found for the salted noodles containing 20% Hylon VII corn starch in the composite flour. The results of the hydrolysis index were converted into estimated glycemic index (EGI) of salted noodles, showing that the highest EGI, 72.8, was in the control sample (Figure 2), followed by 10% HMT (66.8), 20% HMT (63.5), 10% GBF (62.8), and 10% Hylon VII (62.5). Meanwhile, 20% GBF and 20% Hylon VII noodle samples exhibited the lowest EGI values, at 60.5 and 60.1, respectively. A previous research report also indicated that green banana pulp noodles showed lower GI than the control noodles, and this could be credited to the presence of RS and dietary fiber content in the green banana flour [32]. Moreover, previous studies recommended that eating unripe banana flour has a valuable effect on human health because of the high content of RS, dietary fiber, non-starch polysaccharides, and other nutritive and functional components [33,34].

## 4. Conclusions

The incorporation of resistant starch source flour (10–20%, *w*/*w*), for example, the GBF, HMT flour, and Hylon VII flour, with wheat flour generates a dough with appropriate strength and extensible texture, allowing salted noodles to uphold a stiff and elastic form. Moreover, the nutritional value is subsequently enhanced, as the addition of resistant starch source flour with salted noodles resulted in more nutrient components with lower GI value. Furthermore, the 10% GBF noodles exhibited an EGI value of 62.8, as compared to the control sample (72.8). Even though increasing the amounts of resistant starch source flour used caused the cooking characteristics to deteriorate proportionally, supplementing salted noodles with 10% (*w*/*w*) resistant starch source flour (10% GBF) demonstrated equal overall acceptability in terms of sensory evaluation as compared with the control noodle sample.

## Figures and Tables

**Table 1:** Farinograph analysis of wheat flour dough containing different resistant starch source flours.

Composite Flour (%)	Farinographic Dough Characteristics					
	Water Absorption (%)	Dough Development Time (min)	Breakdown Time (min)	Stability (min)	Peak Time (min)	Mixing Tolerance Index (B.U.)
Control	65.6 ^d^ ± 0.1	1.8 ^c^ ± 0.3	23.2 ^a^ ± 0.2	21.0 ^a^	11.2 ^a^	20.0 ^g^
10% GBF	66.2 ^c^ ± 0.1	1.6 ^b^ ± 0.1	16.4 ^b^ ± 0.2	16.0 ^b^	11.5 ^b^	25.0 ^f^
20% GBF	66.4 ^c^ ± 0.1	1.5 ^b^ ± 0.2	13.6 ^c^ ± 0.5	6.5 ^d^	7.7 ^c^	45.5 ^c^
10% HMT	67.8 ^b^ ± 0.1	1.6 ^a^ ± 0.1	12.1 ^d^ ± 0.0	15.0 ^b^	8.1 ^c^	32.0 ^e^
20% HMT	67.6 ^b^ ± 0.1	1.6 ^ab^ ± 0.1	11.4 ^e^ ± 0.4	6.0 ^d^	4.3 ^d^	56.5 ^b^
10% Hylon	68.5 ^a^ ± 0.1	1.6 ^ab^ ± 0.1	10.3 ^e^ ± 0.4	12.5 ^c^	3.1 ^d^	38.0 ^d^
20% Hylon	69.2 ^a^ ± 0.1	1.5 ^ab^ ± 0.1	8.5 ^e^ ± 0.4	5.5 ^d^	1.6 ^e^	63.5 ^a^

^a–g^ Means with different letters within the same column differed significantly (*p* < 0.05) (*n* = 5).

**Table 2:** Extensographic properties of composite flour with different proportions of resistant starch sources at different resting times.

Composite Flour (%)	Extensographic Properties					
	Maximum Resistance to Extension (BU)			Extensibility (cm)		
	45 (min)	90 (min)	135 (min)	45 (min)	90 (min)	135 (min)
Control	58 ^e^	743 ^c^	733 ^e^	16.8 ^a^	13.4 ^a^	13.6 ^a^
10% GBF	668 ^d^	808 ^c^	798 ^d^	14.1 ^b^	13.9 ^a^	12.8 ^b^
20% GBF	696 ^d^	895 ^b^	847 ^c^	13.4 ^c^	12.1 ^b^	10.6 ^c^
10% HMT	780 ^bc^	933 ^b^	959 ^b^	12.7 ^d^	10.8 ^c^	10.7 ^c^
20% HMT	850 ^b^	960 ^b^	>1000 ^a^	11.5 ^e^	10.1 ^c^	9.6 ^d^
10% Hylon	830 ^b^	>1000 ^a^	980 ^b^	11.8 ^e^	10.6 ^c^	9.5 ^d^
20% Hylon	960 ^a^	>1000 ^a^	>1000 ^a^	9.2 ^f^	8.1 ^d^	7.9 ^e^

^a–f^ Means with different letters within the same column differed significantly (*p* < 0.05) (*n* = 5).

**Table 3:** Proximate compositions, total starch content, and resistant starch content of cooked salted noodles containing different proportions of resistant starch source flours.

	Proximate Compositions					
Types of Noodles	Protein	Fat	Ash	Crude Fiber	Total Starch	Resistant Starch
Control	10.25 ^ab^	1.26 ^a^	1.98 ^b^	1.85 ^b^	65.8 ^c^	3.25 ^e^
10% GBF	10.96 ^a^	1.21 ^a^	2.15 ^ab^	2.15 ^a^	65.3 ^c^	7.16 ^c^
20% GBF	11.28 ^a^	1.13 ^bc^	2.31 ^a^	2.12 ^a^	63.5 ^d^	10.92 ^b^
10% HMT	9.53 ^b^	1.18 ^ab^	2.05 ^b^	1.47 ^d^	67.0 ^b^	5.89 ^d^
20% HMT	8.67 ^c^	1.06 ^c^	1.97 ^b^	1.42 ^d^	69.1 ^a^	7.27 ^c^
10% Hylon	9.32 ^bc^	1.15 ^b^	1.93 ^b^	1.84 ^b^	67.2 ^b^	9.83 ^b^
20% Hylon	8.76 ^c^	1.24 ^a^	2.01 ^b^	1.70 ^c^	68.5 ^a^	14.12 ^a^

^a–e^ Means with different letters within the same column differed significantly (*p* < 0.05) (*n* = 3).

**Table 4:** Texture profiles of cooked salted noodles containing different proportions of resistant starch source flour.

	Texture Profile Analysis					
	Hardness (g × force)	Cohesiveness	Springiness	Gumminess (g × force)	Chewiness	Shearing Force (g × force)
Control	525 ^f^	0.62 ^a^	0.92 ^a^	325.5 ^f^	299.5 ^d^	170.2 ^f^
10% GBF	627 ^e^	0.56 ^b^	0.93 ^a^	351.1 ^e^	326.5 ^c^	201.6 ^e^
20% GBF	683 ^d^	0.58 ^b^	0.89 ^b^	396.1 ^d^	352.6 ^b^	239.7 ^d^
10% HMT	612 ^e^	0.62 ^a^	0.87 ^b^	379.4 ^e^	330.1 ^c^	226.3 ^d^
20% HMT	758 ^c^	0.56 ^b^	0.81 ^c^	424.5 ^c^	343.8 ^b^	269.5 ^c^
10% Hylon VII	821 ^b^	0.57 ^b^	0.82 ^c^	468.0 ^b^	383.7 ^a^	287.2 ^b^
20% Hylon VII	947 ^a^	0.57 ^b^	0.72 ^d^	539.8 ^a^	388.6 ^a^	374.5 ^a^

^a–f^ Means with different letters within the same column differed significantly (*p* < 0.05) (*n* = 5).

**Table 5:** The cooking weight gain and cooking weight loss of noodles containing different proportions of resistant starch source flours.

Resistant Starch Source Flour (%)	Cooking Weight Gain (%)	Cooking Weight Loss (%)
0%	95.87 ^a^	1.77 ^b^
10% GBF	92.51 ^a^	2.32 ^a^
20% GBF	90.38 ^b^	2.51 ^a^
10% HMT	95.14 ^a^	2.23 ^a^
20% HMT	92.33 ^a^	2.57 ^a^
10% Hylon VII	85.92 ^c^	2.21 ^a^
20% Hylon VII	81.05 ^c^	2.38 ^a^

^a–c^ Means with different letters within the same column differed significantly (*p* < 0.05) (*n* = 5).

## Data Availability

The datasets used and/or analyzed during the current study are available from the corresponding author on request.
